# Sugar Sweetened Beverages and Weight Gain over 4 Years in a Thai National Cohort – A Prospective Analysis

**DOI:** 10.1371/journal.pone.0095309

**Published:** 2014-05-07

**Authors:** Lynette Lim, Cathy Banwell, Chris Bain, Emily Banks, Sam-ang Seubsman, Matthew Kelly, Vasoontara Yiengprugsawan, Adrian Sleigh

**Affiliations:** 1 National Centre for Epidemiology and Population Health, Australian National University, Canberra, Australia; 2 Sukhothai Thammithirat Open University, Nonthaburi, Thailand; 3 Genetics and Population Health Division, Queensland Institute of Medical Research, Brisbane, Queensland, Australia; Universidad Peruana de Ciencias Aplicadas (UPC), Peru

## Abstract

**Introduction:**

Sugar sweetened beverages (SSBs) are implicated in the rising prevalence of obesity and diet-related chronic diseases worldwide. However, little is known about their contribution to weight gain in Asian populations. This study aimed to investigate weight change associated with SSB consumption between 2005 and 2009 in a large national cohort of Thai university students.

**Methods:**

Questionnaire data were collected from a large Thai cohort (the *Thai Health-Risk Transition: a National Cohort Study*). The analysis was based on responses from 59 283 of the 60 569 (98%) cohort members who had valid SSB consumption and weight variables in 2005 and 2009. The relationship between SSB consumption in 2005 and self-reported weight change was analysed using multiple linear regression models controlled for socio-demographic, activity and (non-validated) dietary factors shown to influence weight.

**Results:**

Higher frequency of SSB consumption in 2005 was significantly associated with greater weight gain between 2005 and 2009 in all age groups and in both sexes (p<0.0001); persons who consumed SSBs at least once a day in 2005 gained 0.5 kg more than those who consumed SSBs less than once a month. The estimated weight gain for the average person in the sample was 1.9 kg (95% C I 1.95–1.96). The difference in weight gain between those who increased their consumption frequency (<once a month to > once per day) between 2005 and 2009 compared to those who maintained it was 0.3 kgs, while persons who reduced their consumption frequency (once a day to > once a month) gained 0.2 kgs less than those whose consumption remained unchanged.

**Conclusion:**

SSB consumption is independently associated with weight gain in the Thai population. Research and health promotion in Thailand and other economically transitioning countries should focus on reducing their contribution to population weight gain and to diet-related chronic diseases.

## Introduction

Population weight gain in Thailand, as elsewhere, poses a looming health and social problem. Thailand has one of the highest frequencies of overweight and obesity in the Asian region [1]; recent Thai National Health Surveys show the age-standardized prevalence of adult obesity (BMI ≥25, Asian cutoff) increased from 25.6% in 1997 to 30.3% in 2004 [2] and 34.7% in 2009, with a substantial female majority [3]. As a result, diabetes, cardiovascular and other diseases are expected to surge [4] accompanied by economic and social costs and health care system challenges [5].

Population weight increase is a complex, multi-factorial problem attributed to socio-economic growth, urbanization, sedentary lifestyles and dietary change [6]. Reversing the structural determinants of weight gain remains a long-term goal but in the interim, targeting reductions in specific calorie-dense foods and beverages for which good replacements exist may be beneficial. One source of ‘useless’ calories is Sugar Sweetened Beverages (SSBs) including carbonated sweet beverages or soda which promote weight gain through their high sugar content, and a low induction of satiety [7], [8].

Thailand is now a major producer of sugar which Thais have consumed in increasing quantities over the last few decades. Between 1969 and 2003 the estimated intake of kilocalories in Thailand increased from 2110 to 2400 [9], and between 1983–2009 sugar consumption almost tripled from 12.7 to 31.2 kilograms per person per year [10]. Consumption of SSBs, which contain high fructose corn syrup and/or sucrose, has played a substantial part in this increase in Thailand. The most recent National Health Examination Survey in Thailand (2009) reported that over 30% of adolescents aged 6–14 years consumed SSBs almost every day or more often, as did 16% of those aged over 15; relative increases of 50% and 100% since the previous survey in 2003 [3].

While the evidence on the causal relationship between SSBs and weight gain in adults is firming [11], much of it is unhelpful for assessing causality (many studies being too small, too brief or at risk of reverse causality) so it has been challenging to grasp the true nature of the association. A very recent WHO-sponsored review by Te Morenga et al, [12] restricted to the few relevant prospective cohort studies of reasonable duration and randomised trials (RCTs) of adequate design, concludes that SSBs are likely determinants of adult weight gain, despite the still relatively limited data. One prospective study [13] has linked SSB use to modest weight gain in an older Asian population, but the relationship was not examined in detail. In this paper, we add to the available data in general, and substantially expand knowledge in the Asian context, via investigating level of consumption of SSBs in a large national cohort of Thai adults in 2005 and 2009 and their contribution to weight gain over this time.

## Methods

### Ethics Statement

Ethics approval was obtained from Sukhothai Thammathirat Open University Research and Development Institute (protocol 0522/10) and the Australian National University Human Research Ethics Committee (protocols 2004344 and 2009/570). Informed written consent was obtained from all participants. Data were de-identified before analysis.

### Study population

This study uses data collected in a large Thai cohort (the *Thai Health-Risk Transition: A national Cohort Study*). In 2005, 200 000 adult Sukothai Thammithirat Open University (STOU) students residing throughout Thailand were mailed a consent form, a reply-paid envelope and a 20 page questionnaire covering socio-economic, demographic, cultural and lifestyle characteristics, health-risk behaviours and health outcomes. Completed questionnaires were returned by 87 134 (44%) students aged between 15–87 years [14], [15]. Following this, efforts were made to feedback information to cohort members and to maintain contact; including checking the cohort data base against the STOU student database and identifying deaths by linking citizen ID numbers of the 99% cohort members who provided them to the national death register. In 2008–9, a 12 page follow-up questionnaire was mailed out and returned by 70% (60 569) of cohort members after four mail-outs and additional phone calls [15]. Analysis was carried out on the 59 283 cohort members with valid SSB consumption and weight variables at both surveys.

### Exposure assessment

The primary exposure under examination was SSB consumption in 2005; change in its consumption frequency between the two surveys was the secondary study exposure. Frequency of SSB consumption (translated in the Thai survey as soda or carbonated sweetened beverages without distinguishing from diet soft drinks), was reported by categories, ranging from less than once a month to once a day or more. To maintain consistency with research literature the term SSBs is used in this paper even though our survey question asked about soda (see [13]).

### Outcome assessment

Weight gain, rather than BMI, is the outcome measure as it more sensitive to change over a short time period such as the 4 years in this study. Height was not expected to change. Relevant measures reported in 2005 included self-reported height in centimeters (cm) and weight in kilograms (kg), measured without shoes; both were shown to be recorded accurately enough for use based on a comparison of self-report and independent measures taken with separate sample of 750 students from STOU [16]. BMI was derived from the ratio of a person's weight divided by the square of the height in meters and recorded in kg/m^2^. Asian cut-points were used to define BMI categories classifying adults as underweight (<18.5 kg/m^2^); normal (18.5 to <235 kg/m^2^); overweight-at-risk (23–24.9 kg/m^2^) or obese (25 kg/m^2^ or over). These measures were repeated in 2009.

### Assessment of covariates

Self-reported urban or rural residence in 2005 and when aged 10–12 years old was used to create four life-course urbanization categories: rural to rural (RR), rural to urban (RU), urban to rural (UR), and urban to urban (UU). Highest educational level achieved was classified into high school graduation, post-secondary or diploma level and university graduation.

Risk factor measures included tobacco and alcohol consumption (current or not) and hours per day of leisure time, physical activity, screen time, and sitting for any purpose. Self-reported housework and gardening were categorized into 5 groups ranging from seldom or never to 3 or more times a week. Frequency of fried food consumption and western fast food were reported by categories, ranging from less than once a month to once a day or more. In 2008–9, these measures were repeated for height, weight, BMI, location of residence and other health risks including SSB consumption. The data were extensively checked with SQL and SPSS software after scanning and digitizing.

### Statistical analysis

The relationship between weight change and SSB consumption was analysed using multiple linear regression models. All models controlled for socio-demographic factors (age, sex, location of residence, urbanization status, education, marital status), smoking, drinking and baseline BMI. Subject matter knowledge was used to identify a range of relevant variables on physical activity (strenuous, moderate or mild exercise, walking, housework/gardening, sleeping, screentime (TV or computer) and sitting time) and diet (foods with coconut milk, deep fried food, fermented, roasted, uncooked, instant and canned foods, milk, soy products, Western-style fast-food, fruit and vegetables) which were systematically examined for their relationship with weight gain. The final models included three physical activity variables (leisure time physical activity in the form of weighted number of sessions, hours of housework or gardening and hours of screentime) and two energy-dense diet variables (fried food and Western style fast food).

A variety of models were fitted which controlled for the effect of physical activity and diet as measured in 2005 and in 2009; likelihood ratio tests were used to choose between models. The main effects and interaction of SSB consumption in 2005 and in 2009 were modeled to examine the effect of change in SSB frequency on weight change. The analysis was performed with STATA 12 and based on 59 283 of the 60 569 (98%) cohort members who had valid SSB consumption and weight variables at both surveys. The analysed group were compared to the 26 565 members of the initial cohort who did not respond to the follow-up questionnaire to assess the potential for selection bias; details and results are provided below in the limitations section of the Discussion.

## Results

Respondents to both surveys were broadly representative of the Thai population on socio-economic, demographic (other than age) and ethnic characteristics. Of the 59 283 who returned questionnaires in 2005 and 2009, 54.8% were females. The median age was 30; 51.5% were urban residents, and the median annual income was $US 2550.

### Patterns in SSB consumption

Exposure to SSBs and weight, BMI and obesity levels in 2005 and 2009 are broken down by sex in Table 1, demonstrating that most people consumed SSBs 3 times a month or less (55% in 2005, 65% in 2009), and only a minority drank them every day (7% in 2005, 5% in 2009; Table 1). The calculation of the sample's mean weight change was based on an average of individual weight change calculations. Overall, mean weight increased 1.9 kg (SD 4.3); from 52 kg to 54.2 kg for females (an increase of 1.8 kg) and from 65 kg to 67.1 kg for males, (an increase of 2.0 kg) with a corresponding increase in the prevalence of female obesity from 10.4% to 15. 6%; male obesity from 23.9% to 29.9%; and for both 16.5% to 22.1% (Table 1).

**Table 1:** SSB consumption and body size measures in 2005 and 2009 (n = 59,283).

	Female		Male		Total	
	2005	2009	2005	2009	2005	2009
SSB consumption, %						
Once a day or more	7	5	7	5	7	5
3–6 times per week	14	10	19	14	16	11
1–2 times per week	20	16	25	21	22	19
1–3 times per month	29	28	28	31	29	30
Never or less than once a month	30	41	21	29	26	35
Weight, kg (mean (SD))	52.0 (8.8)	54.2 (9.6)	65.0 (10.1)	67.1 (10.7)	58.1 (11.4)	60.0 (11.9)
Weight gain 2005–2009, kg (mean (SD))	1.8 (4.5)		2.0 (4.1)		1.9 (4.3)	
BMI, kg/m^2^ (mean (SD))	21.0 (3.2)	21.8 (3.6)	23.0 (3.2)	23.7 (3.3)	21.9 (3.4)	22.7 (3.6)
Obese, %	10.4	15.6	23.9	29.9	16.5	22.1

(Mean weight gain based on an average of individual weight change calculations).

A heavier SSB consumption pattern of at least three times a week was more frequent in males, in younger persons, in those living in Bangkok or in those who had lived in urban areas for longer, and in those who had less education, were single, or who smoked or drank alcohol (Table 2). These socio-demographic patterns of SSB consumption were similar in 2005 and in 2009 (Table 2).

**Table 2:** Characteristics of those consuming 3 or more SSBs per week and weight change between 2005 and 2009.

		% Consuming SSB ≥3 times per week		% with unchanged SSB levels in 2005 and 2009	Weight change, 2005 to 2009 (kg)	
	n	2005	2009		mean	(SD)
	59,283	23	16	42	1.9	(4.3)
						
Males	32,488	26	19	44	2.0	(4.1)
Females	26,795	20	14	40	1.8	(4.5)
						
<25	13,177	28	23	37	2.4	(4.8)
25 to <35	26,366	25	18	41	2.3	(4.3)
> = 35	19,740	16	10	46	1.2	(3.7)
						
Bangkok	9,906	27	21	43	1.9	(4.3)
Urban, not Bangkok	21,452	23	17	42	2.0	(4.3)
Rural	27,705	21	15	41	1.9	(4.3)
						
Always urban	26,063	29	22	42	1.9	(4.2)
Urban to rural	18,097	27	21	42	1.9	(4.1)
Rural to urban	2,615	22	15	40	2.0	(4.7)
Always rural	12,255	20	14	42	2.0	(4.5)
						
High school	27,347	24	18	41	1.8	(4.3)
Diploma	15,959	23	17	41	2.2	(4.3)
University	15,841	20	14	44	2.0	(4.2)
						
Single	46,217	24	18	42	1.9	(4.2)
Partnered	12,926	22	16	42	2.2	(4.4)
						
Current smoker	6,323	31	21	42	1.9	(4.2)
Not current smoker	51,462	22	16	39	1.9	(4.7)
						
Current drinker	27,765	26	18	43	1.9	(4.2)
Not current drinker	30,815	20	15	40	2.0	(4.3)

(Age, location of residence, urbanisation type, education, marital, smoking and drinking status in 2005).

**Figure 1:**
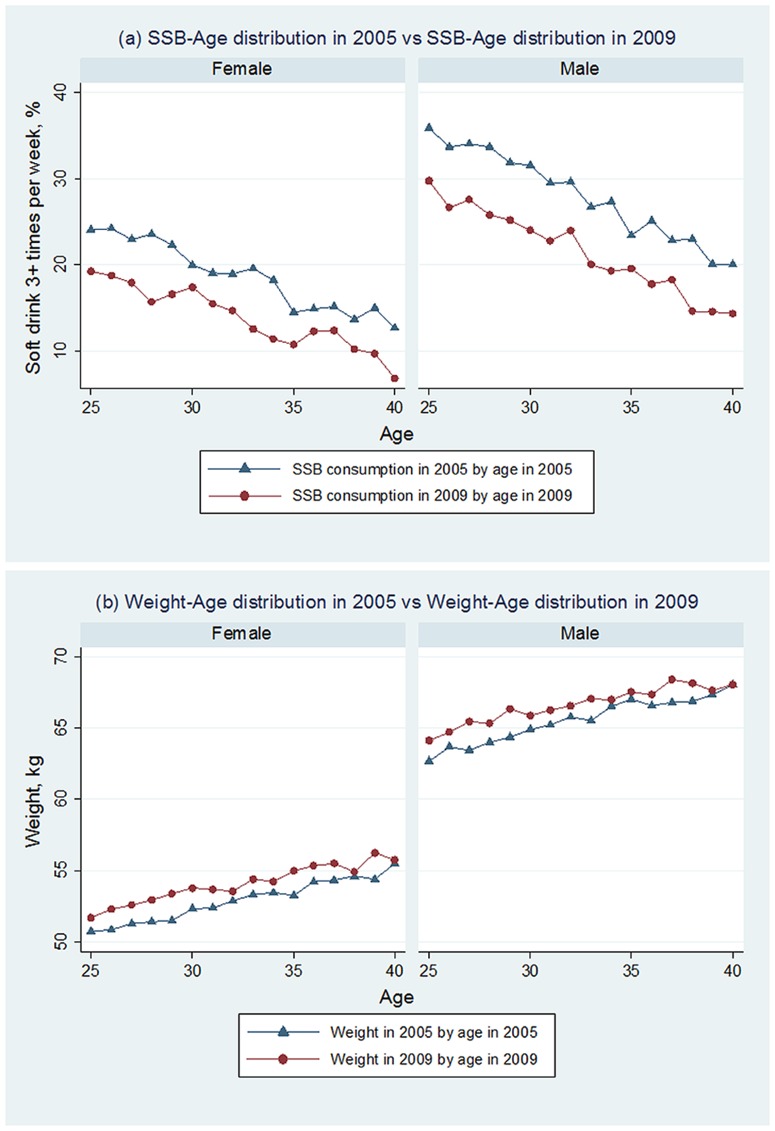
SSB and age and age-weight distribution in 2005 and 2009.

Just under half the cohort (37% to 46%) continued to maintain the same level of SSB consumption in the period between the two surveys, although overall, SSB consumption declined. The proportion of persons consuming SSBs three or more times per week dropped from 23% in 2005 to 16% in 2009 (Table 2). This decline is not an artefact of the cohort ageing. Figure 1a, which compares SSB consumption in the two surveys for persons of the same age, illustrates clearly that consumption has declined by about the same amount in all ages and in both sexes.

### SSB consumption in 2005 and weight change 2005–2009

Figure 1b compares weights in the two surveys for persons of the same age. For both sexes mean weight at every age was higher in 2009 than in 2005 indicating that weight was increasing in the population as a whole. Females, older persons, high school graduates and single persons showed less increase in weight than their comparators (Table 2).

SSB consumption in 2005 was strongly predictive of weight change between the 2005 and 2009 surveys, with increasing consumption frequency statistically significantly associated with greater weight gain in all age groups and in both sexes. Effect modification was modest or absent for most other variables (Table 3). These associations remained after adjusting for baseline socio-demographic factors, smoking and drinking, physical activity (leisuretime, physical activity, screentime and housework/gardening) and consumption of energy-dense foods (deep fried foods and Western-style fast foods).

**Table 3:** SSB consumption in 2005 and weight change between 2005 and 2009, by age and sex.

	Weight change between 2005 and 2009, kg *[mean (SD)]*													
	<25				25 to <35				35+				All	
	Males		Females		Males		Females		Males		Females			
SSB consumption in 2005														
Once a day or more	3.4	(5.1)	2.6	(5.0)	2.4	(5.0)	2.5	(4.4)	1.3	(4.1)	1.6	(3.4)	2.4	(4.7)
3–6 times per week	3.5	(5.1)	2.2	(4.3)	2.4	(4.6)	2.3	(4.3)	1.1	(4.0)	1.8	(3.3)	2.2	(4.4)
1–2 times per week	3.1	(5.3)	2.1	(4.3)	2.2	(4.4)	2.4	(4.2)	1.0	(3.9)	1.6	(3.4)	2.1	(4.3)
1–3 times per month	3.0	(5.2)	1.9	(4.6)	2.2	(4.3)	2.3	(4.1)	0.9	(4.0)	1.5	(3.3)	1.9	(4.2)
Never or less than once a month	2.5	(5.8)	1.7	(4.7)	2.0	(4.5)	2.2	(4.1)	0.7	(3.7)	1.4	(3.3)	1.6	(4.1)
*Test for trend*	*0.003*		*<0.0001*		*<0.0001*		*<0.0001*		*<0.0001*		*<0.0001*		*<0.0001*	

Note: These associations remained after adjusting for baseline socio-demographic factors, smoking and drinking, physical activity (leisuretime, physical activity, screentime and housework/gardening) and consumption of energy-dense foods (deep fried foods and Western-style fast foods).

SSB consumption in 2005 was the strongest predictor of future weight gain among the physical activity and energy-dense diet variables available in our survey (Table 4). In the model, persons who consumed SSBs at least once a day gained 0.5 kg more than those who consumed SSBs less than once a month, slightly less than the overall unadjusted effect of 0.8 kgs (2.4 kgs once a day to 1.6 kgs for less than once a month) (Table 4).

**Table 4:** SSB consumption in 2005 and weight change between 2005 and 2009.

		Weight change, kg		Model^a^			
	n	*mean*	*(SD)*	*wt. ch.*	*95% CI*		*p*
**SSB consumption in 2005**							
Once a day or more	3,857	2.4	(4.7)	0.5	0.3	−0.6	<0.0001
3–6 times per week	9,408	2.2	(4.4)	0.3	0.2	−0.5	<0.0001
1–2 times per week	13,141	2.1	(4.3)	0.2	0.1	−0.4	<0.0001
1–3 times per month	16,955	1.9	(4.2)	0.1	0.0	−0.2	0.004
Never or < once a month	15,425	1.6	(4.1)	0			
*p (Trend)*							*<0.0001*
**Leisure-time physical activity in 2005, no. sessions**							
0	4,255	1.9	(4.3)	−0.1	−0.3	−0.1	0.2
1–7	22,364	2.0	(4.3)	−0.1	−0.2	−0.0	0.2
8–14	17,085	2.0	(4.2)	0.0	−0.1	−0.1	0.5
15+	11,767	1.9	(4.4)	0			
*p (Trend)*							*0.1*
**Screen time in 2005, no. hours**							
8+	1,647	2.1	(4.9)	−0.1	−0.3	−0.2	0.6
5–7	6,810	2.1	(4.7)	0.0	−0.2	−0.1	0.6
3–4	20,899	2.0	(4.3)	0.0	−0.1	−0.0	0.4
0–2	28,900	1.8	(4.1)	0			
*p (Trend)*							*0.4*
**Housework or gardening in 2005**							
Seldom or never	3,304	1.9	(4.8)	0.0	−0.2	−0.2	1.0
1–3 times per month	6,423	2.0	(4.4)	0.1	0.0	−0.3	0.03
1–2 times per week	15,961	2.1	(4.3)	0.1	0.0	−0.2	0.04
3+ times per week	32,837	1.9	(4.2)	0			
*p (Trend)*							*0.1*
**Fried food consumption in 2005**							
3+ times per week	30,883	2.1	(4.4)	0.1	−0.2	−0.3	0.6
1–2 times per week	18,288	1.9	(4.2)	0.0	−0.3	−0.2	0.8
1–3 times per month	8,137	1.8	(4.1)	0.0	−0.3	−0.3	1.0
Less than once per month	1,442	1.6	(4.1)	0			
*p (Trend)*							*0.078*
**Western-style fast food in 2005**							
3+ times per week	132	2.6	(4.7)	0.4	−0.4	−1.2	0.3
1–2 times per week	2,222	2.2	(5.0)	0.1	−0.1	−0.3	0.4
1–3 times per month	10,954	2.2	(4.4)	0.1	0.0	−0.2	0.003
Less than once per month	44,868	1.9	(4.2)	0			
*p (Trend)*							*0.007*

a Regression model of weight change on SSB consumption in 2005 controlling for socio-demographic factors including baseline BMI (not shown) and physical activity and diet variables measured in 2005.

The associations between SSB consumption in 2005 and weight change persisted even when physical activity factors and energy-dense diet as measured in 2009 were included in the model (data not shown). In Table 4 the regression coefficients represent weight change in a given category relative to the reference category of that variable. The only other relevant variable in the model that showed a significant trend was western style fast food consumption in 2005 (p 0.007). The estimated weight gain for the average person in the sample was 1.9 kg (95%C I 1.95–1.96).

### Change in SSB consumption between 2005 and 2009 and weight change 2005–2009

**Figure 2:**
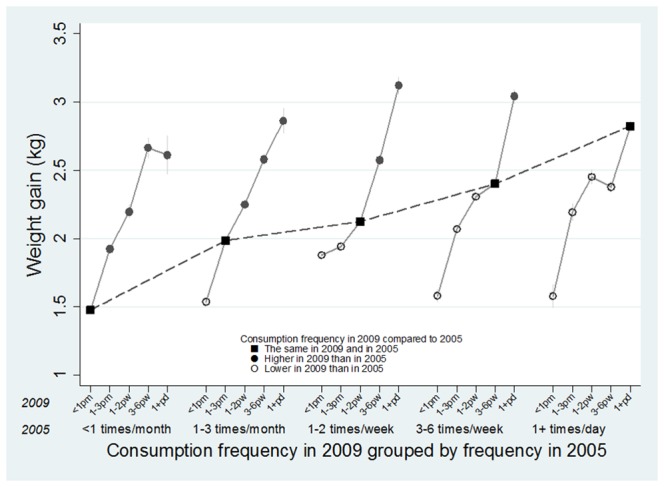
Estimated weight gain by SSB consumption frequency in 2005 and 2009. Estimates were derived from a regression model adjusting for socio-demographic characteristics and lifestyle factors (smoking, drinking, physical activity and energy-dense diet).

Only 20% of persons reported an increased frequency in SSB consumption while 38% reported a decreased frequency between the two surveys and most (42%) reported the same frequency of SSB consumption. Males, older persons, university graduates, smokers and alcohol drinkers were more likely to report no change in SSB consumption frequency (Table 2). Figure 2 shows the weight changes (kgs on the Y axis) estimated for the five combinations of SSB consumption which range from less than once a month to once a day (X axis) at the two time points controlling for socio-demographic characteristics and lifestyle factors (smoking, drinking, physical activity and energy-dense diet in 2005 and 2009). Confidence intervals, shown as vertical grey lines, are narrow for some subgroups due to the large study size and are therefore difficult to see. SSB consumption in both 2005 and in 2009 were statistically significant factors in the model (LR test comparing model with consumption in 2005 and 2009 vs model omitting 2005 consumption: p<0.0001). Overall the average person in the sample gained 1.9 kg (mean SD 4.3). In persons who maintained the same frequency of consumption at the two surveys, weight gain increased steadily (<once/month: 1.5 kg, 1–3/month: 2.0 kg, 1–2/week: 2.1 kg, 3–6/week: 2.4 kg, daily+: 2.8 kg). Due to this study's large sample size, confidence intervals are only operational at the second decimal point rendering them unnecessary. The figure also shows the weight change that occurred for each consumption category as people's consumption varied between 2005 and 2009. For example, people who drank once to 3 times a month in 2005 and increased their consumption to once a day in 2009 increased their weight by almost 3 kgs. In contrast, if their consumption dropped to less than once per month their weight increase was smaller (1.5 kgs). The 20% of men and women who increased their consumption showed consistently greater weight gains compared to persons who maintained the same level of consumption while those who decreased their intake showed consistently less weight gain (Figure 2). The excess average weight gain for those who increased their consumption of SSBs compared to those who maintained it was 0.3 kgs, while persons who decreased their consumption gained 0.2 kgs less than the stable group.

## Discussion

This large, prospective cohort study of Thai adults provides detailed information on patterns of association between drinking SSBs and subsequent weight gain in Asia. More frequent SSB consumption in 2005 was associated with weight gain between 2005 and 2009 among men and women of all ages, independent of other risk factors. The weight of men who drank SSBs at least three times a week in 2005 increased on average by 2.0 kgs between 2005 and 2009 while the weight of women who consumed in a similar fashion increased by 1.8 kgs. Persons who drank SSBs at least once a day over the 4 years gained on average 0.5 kg more than those who consumed it less than once a month. Weight gain was accelerated in those who increased their intake during follow-up and slowed among those who lowered it. Men, those under 25, urban dwellers, Bangkok residents and those with lower levels of education were likely to consume SSBs more than three times a week. They were also more likely to smoke and drink suggesting that they did not adopt health promotion messages in general.

A current, definitive WHO-sponsored review with a strong public health focus by Te Morenga [12] presents results from prospective cohorts (minimum 1-year follow-up) and RCTs (minimum duration 2 weeks) which assess relations between sugar intake (or removal in RCTs) and measures of body fatness. The subsets which address our central question regarding SSB intake in adults were relatively small (6 papers reporting 7 prospective cohort studies of SSBs with 2–30 years of follow-up; 5 RCTs (lasting 2.5 to 10 months) studying reduced sugar intake, 10 (8 running for less than 8 weeks) adding sugars to the diet). The analyses of cohort data by Te Morenga, (details and figures on the Web, [12]) show fairly consistent positive associations of higher body weights with baseline SSB intake and with increases in SSBs. There is no summary overall estimate to compare directly with ours. The trial data [12] show short-term reductions in sugar intake led to significantly reduced weight overall (−0,8 kg, 95% CI −1.2 to −0.4); trials with added sugars (4 as SSBs) yielded an almost identical increase (0.72, 95% CI 0.3 to 1.2). The 2 longer-term (>8 weeks) trials of added sugars reported much larger increases, 2.7 kg, 95% CI 1.7 to 3.8. The 5 cohort studies in children included in the meta-analysis all showed increases in fatness over varied time periods among those consuming higher amounts of SSBs, OR  = 1.6 (1.3–1.8) (34). It has been harder to reproduce the weight benefits of reducing sugars in RCTs in children, with poor compliance being a major challenge. Where this has been met in the longer term (a year or more), results are solidly suggestive of benefit [17], [18], [19], [20].

In the only other large cohort study of Asian adults (older Chinese Singaporeans), participants who consumed SSBs 2 to 3 times a week had a significant increase in weight (0.53 kg over 5.9 years) compared with those who drank less than monthly [13], quite similar to our estimate; no further details were reported. Added to the above findings, our results strengthen and extend the evidence, for Asia in particular as well as in the wider sphere, that drinking SSBs in even moderate amounts leads in the mid-to-longer term to increases in weight; that increasing intake increases this effect; and perhaps most importantly, that diminishing it reduces weight gain. There are good reasons for accepting these observations as reflecting the causal link proposed by Hu (11). While it is difficult to put an exact figure on the size of the obesogenic effect of drinking SSBs, it is clearly substantial and continuing over time; there is high consistency in the effect in both cohort studies and RCTs (where confounding by other dietary factors is avoided), including lowered risk with decreased consumption in trials and in our cohort and others [21], [22]; associations are greater at higher intakes; directionality of effect is clear. There is an obvious mechanism of adding ‘empty’ calories with limited effect on satiety [7] with no compensatory reduction in overall energy intake in contrast to the ingestion of solid sugars which induce a compensatory reduction in energy intake [7]. Thus targeting SSB consumption as an accessible lever in public health campaigns to control weight gain in both children and adults, in Thailand and elsewhere, makes good sense [11]. Of course simply identifying the lever is insufficient in itself, but these products stand out from many other energy-providing components of our diets as being more readily substitutable, and thus potentially more amenable to control through a mix of personal and social interventions.

In light of the evidence demonstrating the public health benefits of reducing SSB consumption it is heartening to note that in contrast to increasing SSB consumption in Thailand [3] and internationally, the proportion of the Thai cohort consuming SSBs three or more times a week declined from 23% in 2005, to 16% in 2009 (independent of age and sex) with only 20% reported increased frequency of consumption. Thailand has Asia's largest per capita consumption of carbonated beverages at 39.2 litres [23]. A business report observed that the SSB market has maintained a 6% growth even while sales of bottled water, fruit/vegetable juice and other drinks [24] increased. Thailand has campaigned since 2002 to reduce children's sugar consumption [25] and is raising awareness among adults of the health risks of sugar and SSBs [26]; a message that may be more likely to be picked up by TCS members than the less educated general population.

Generally, in western countries SSB consumption has increased only slightly over the last several decades but appears now to be slowing. Recent business reports show that Australia's consumption of soda has declined slightly since 2005 [27], recent increases in SSBs in the UK were a modest 0.7% [28] decreases are occurring in the US [29] with a Wall Street Journal expressed concern about declining US soda sales [30]. However, consumption of other types of SSBs such as sports and energy drinks may have increased among adolescents [31].

One limitation of this study was that we only recorded self-reported frequency of consumption so it is not possible to estimate the contribution of SSBs to total energy intake as an explanation for the observed association. However, using a recent estimate of average Thai daily energy consumption as 3100 kcal per person [32], and a 12 oz can of soda as a serve (150 kcal see [8]), we estimate SSBs contributed 4.83% of the daily energy consumption to those who drank them daily.

A second limitation is that our survey question used in 2005 was “How often do you consume soda?” This question was not part of a validated food frequency questionnaire and it is less inclusive than a question about SSBs more broadly; it does not capture consumption of sweetened teas, flavoured milk and sports drinks contributing to an under-estimation of sugar consumption from SSBs. Nor did we specifically ask about artificially sweetened or diet drinks; however, these are known to make up only about 1 to 3% of the Thai SSB market [33], [34]. It is likely that SSB intake is, if anything, under-reported overall due to the use of this single question. Self-reported weight is known to be quite accurate in our cohort [16], so overall it is unlikely that the relations we observed were exaggerated by measurement error; if anything, the exposure misclassification will have had the opposite effect.

A third limitation is loss to follow-up although the rate (32% = 27 851/87 134) was reasonable for a current large-scale observational study. There were some minor differences between respondents and non-respondents. We have no direct measure of whether non-response could be related to the outcome of interest (i.e. weight change) [35], [36], but the prevalence of obesity among respondents at baseline (17%) and non-respondents (14%) was similar. The main reason for non-response was loss of follow up contact with younger and more mobile cohort members; 52% of respondents were aged 30 or younger compared with 72% of non-respondents; and daily SSB consumption was somewhat lower among respondents than non-respondents (23% to 30%). As well as age there are a number of other covariates which were related to some degree to both non-response and outcome (marital status, urbanisation type, location of residence, education, smoking and fried food consumption); their inclusion in the regression model will have further mitigated the potential for selection bias to affect results materially. The possibility of residual confounding by other dietary components has to be considered given the limitations in our dietary data and the mild confounding of the unadjusted estimates indicated by our multivariable results. This will of course have been offset at least in part by the exposure misclassification.

A final limitation is that cohort members are more educated and somewhat younger than the general Thai population [14]; however, associations and trends identified among the cohort are generally consistent over time and may be manifested among the wider Thai population in the near future. This study derives from a large sample size drawn from diverse economic, social and geographic backgrounds and is similar to the STOU student body from which it is drawn. It is only the second study known to us conducted among an Asian population vulnerable to the effects of weight gain, type 2 diabetes, metabolic syndrome and other diet-related chronic diseases. Adding to the importance of this study is the growing body of evidence linking SSB consumption directly with these conditions [7], [8] particularly in vulnerable Asian populations [13] and among Thais who are comparatively short statured [37] and more prone to metabolic challenge.

The decrease in SSB consumption in the Thai cohort may signal future directions for the Thai population, particularly in light of Thailand's campaign to reduce sugar consumption. Thailand, which is a model for progressive food and nutrition policy [38], [39], has already restricted advertising and sales of sugar products to children. Nevertheless, successful SSB-lowering trials in children and adults elsewhere offer examples for further large scale public health interventions in Thailand; for example combining different approaches to SSB reduction at the Thai province or district level for comparison. International trials [17], [19] also illustrate the need for continued public health efforts to maintain reduced SSB consumption and weight over time. Nevertheless, SSBs should not be the only focus of health promotion; their consumption often co-occurs with low physical activity levels and energy-dense diets which are independently associated with weight gain. Indeed not only should reductions be made in the population's energy consumption via diet but environmental changes to increase the populations' use of energy should be encouraged [40], [41].

## Conclusion

Sugar consumption, particularly in SSBs, is now considered to be a major health threat comparable to smoking [38] and evidence suggests that removing SSBs from diets will have a positive impact on weight over time. They increasingly are a target for health promotion because they have no redeeming nutritional benefits and can be eliminated from the diet without ill effects. Within our Thai cohort we will continue to monitor SSB consumption and links with diabetes, metabolic syndrome and dental health and we will increase the precision of our capture of SSBs; we note the desirability of other longitudinal studies doing the same. As a key ‘transitional’ consumable, further study of the economic and socio-cultural trends related to SSB consumption in Thailand will help illuminate the country's health and nutrition transition.
